# TRPV4: A Physio and Pathophysiologically Significant Ion Channel

**DOI:** 10.3390/ijms21113837

**Published:** 2020-05-28

**Authors:** Tamara Rosenbaum, Miguel Benítez-Angeles, Raúl Sánchez-Hernández, Sara Luz Morales-Lázaro, Marcia Hiriart, Luis Eduardo Morales-Buenrostro, Francisco Torres-Quiroz

**Affiliations:** 1 Departamento de Neurociencia Cognitiva, División Neurociencias, Instituto de Fisiología Celular, Universidad Nacional Autónoma de México, Mexico City 04510, Mexico; mbenitez@ifc.unam.mx (M.B.-A.); rsanchez@ifc.unam.mx (R.S.-H.); saraluzm@ifc.unam.mx (S.L.M.-L.); mhiriart@ifc.unam.mx (M.H.); 2 Departamento de Nefrología y Metabolismo Mineral, Instituto Nacional de Ciencias Médicas y Nutrición Salvador Zubirán, Mexico City 14080, Mexico; luis.moralesb@incmnsz.mx; 3 Departamento de Bioquímica y Biología Estructural, División Investigación Básica, Instituto de Fisiología Celular, Universidad Nacional Autónoma de México, Mexico City 04510, Mexico; ftq@ifc.unam.mx

**Keywords:** TRP channels, TRPV4, structure, disease

## Abstract

Transient Receptor Potential (TRP) channels are a family of ion channels whose members are distributed among all kinds of animals, from invertebrates to vertebrates. The importance of these molecules is exemplified by the variety of physiological roles they play. Perhaps, the most extensively studied member of this family is the TRPV1 ion channel; nonetheless, the activity of TRPV4 has been associated to several physio and pathophysiological processes, and its dysfunction can lead to severe consequences. Several lines of evidence derived from animal models and even clinical trials in humans highlight TRPV4 as a therapeutic target and as a protein that will receive even more attention in the near future, as will be reviewed here.

## 1. Introduction

Ion channels are proteins that participate in multiple cellular functions including the generation of electrical signals in the nervous and muscular systems, in the transport of electrolytes, the secretion of hormones, etc. Some of these ion channels work as receptors to changes in temperature, mechanical stimuli, osmolarity, and acidity [1,2,3]. Among these are members of the Transient Receptor Potential (TRP) superfamily that are characterized by being weakly voltage-dependent nonselective cation channels. The importance of the more of 30 TRP channels described to date is well exemplified by their roles in physiology including phototransduction in invertebrates [4,5,6], responses to painful stimuli and to temperature [7,8,9] and to intracellular Ca^2+^ store depletion [10,11], modulation of the cell cycle [12,13] and regulation of the function of several organs such as the pancreas, lung, kidney, etc. [14].

The general structure of TRP channels, resolved by single-particle cryo-electron microscopy, for several members of this family of proteins shows the presence of intracellular amino (N)- and carboxyl (C)-terminal regions and six (S1–S6) transmembrane domains, where S5–S6 give rise to the pore or ion conduction pathway [15].

Each of these regions in the channels plays a role in the changes associated to their activation and their ability to alternate among conformations, allowing these ion channels to regulate several cellular functions. Thus, it is important to understand the molecular details of the modulation of these proteins by several types of stimuli that are relevant during physiological and pathophysiological contexts.

In this sense, important roles for several TRP channels have been associated to various diseases [16,17,18,19,20]; however, there is still quite a bit of work to be done since the roles of some of these proteins in physiology are still being uncovered. It is important to note that, while some TRP channels have been extensively studied (i.e., TRPV1, the first mammalian TRP channel to be cloned) [7], other members of this family of ion channels have proven to be somewhat more elusive.

This review will focus on the TRPV4 channel for which only few endogenously produced agonists have been described, although it is clearly a physiologically important protein. This channel has been shown to regulate the homeostasis of intracellular calcium concentrations [Ca^2+^]_i_ [21] participating in the integrity of osmoregulation [22,23], endothelial barriers [24,25,26], control of vascular tone [27], nociception [28], bone homeostasis [29], pulmonary [30,31], and renal function [23], as well as in itch [32]. In fact, several mutations in TRPV4 have been associated to congenital diseases, some of which will be reviewed and discussed here and we will mention recent discoveries in this rapidly changing field of study.

## 2. General Properties and Structure of TRPV4

Described for the first time in the year of 2000, TRPV4 has acquired the following names according to the functional features observed when it was studied: OSM-9 [33], VRAC or VR-OAC [22], VRL-2 [34], and TRP12 [35].

TRPV4 is expressed in smooth muscle cells in the pulmonary aorta and artery, brain arteries [36,37], vascular endothelium [38,39,40], epidermal keratinocyte cells [41], epithelia of the trachea and lungs (specially in cilia of the bronchial epithelium) [42], Fallopian tubes [43], ciliated epithelia of bile ducts [44], epithelial cells of the human cornea [45], insulin secreting β cells of the pancreas [46], epithelial cells of the urinary system [47], urothelial cells in the renal pelvis, ureters, urethra, urinary bladder [48,49], primary afferent sensory neurons that innervate the gastrointestinal tract [50], as well as in enterocytes and enteroendocrine cells [51,52], etc.

TRPV4 is a nonselective cation channel with higher permeability for Ca^2+^ (due to interaction of the ion with residues D672, D682, and M680 in the pore region) than for Mg^2+^ as compared to Na^+^ [53,54] and that has been described to be both an inward and outward rectifier depending on whether Ca^2+^ is absent or present [22,53]. In fact, this channel has been shown to be inhibited or potentiated in a Ca^2+^ concentration-dependent fashion [55], a process that has been described to rely on the presence of residue F707 [56].

Like many other TRP channels, TRPV4 is a polymodal protein activated by temperatures around 27 °C, hypoosmotic conditions, and mechanical stress [22,23], and blocked or antagonized by GSK3527497 [57], GSK205 [58], derivates of GSK205 [28], ruthenium red [59], and Gd^3+^ (which has also been shown to block stretch-activated channels in bacteria) [58,60], RN-1734 [61], and RN-9893 [62]. Ligands for this channel include plant chemicals such as bis-andrographolide from *Andrographis paniculate* [63]; phorbol derivatives (i.e., 4α-phorbol 12,13-didecanoate or 4αPDD [53]), the flavonoid apigenin [64], and the widely used synthetic agonist GSK1016790A [65]. Endogenous agonists or activity modulators of TRPV4 are phosphatidylinositol-4,5-biphosphate (PIP_2_), which binds to the ^121^KRWRK^125^ region and allows for activation by temperature and osmotic changes [66]; 5,6-epoxyeicosatrienoic acid (5,6-EET), a product of the metabolism of arachidonic acid through cytochrome P450, which binds to residue K535 [67,68] of the channel activating it, and polyunsaturated fatty acids that affect the activity of the channel through changes in membrane fluidity [69].

A structure for the *Xenopus tropicalis* TRPV4 channel was recently resolved from a nearly complete protein (amino acids 133–797) in which only a few residues from the N- and C- termini were deleted and a glycosylation site was modified (N647Q) [70]. Deng and collaborators found that the TRPV4 channel is a symmetric tetramer formed by subunits with six transmembrane domains with linking loops that resemble those of voltage-gated ion channels (VGICs), where S1–S4 domains encircle the pore formed by S5–S6 [70].

**Figure 1:**
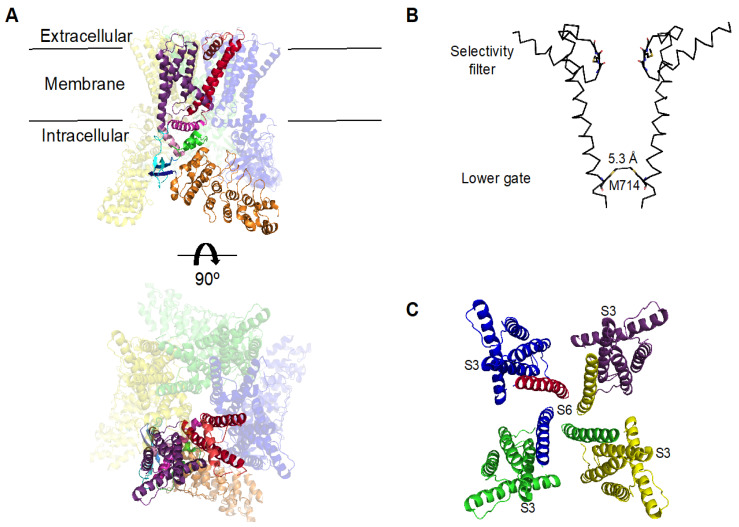
Structure of the TRPV4 channel. (**A**) Frontal view (top) and extracellular view (bottom) where one single subunit of the tetramer and each of its domains are shown in a different color (ARD in orange, β1 and β2 in blue, Helix Turn Helix in green, PreS1 in pink, S1–S4 in purple, S5–S6 in red, TRP box in magenta, and β3 in cyan). The membrane boundary is delimited by the black lines. (**B**) TRPV4 pore diameter. The structures of S6 and the pore helix of two subunits are shown. The distance between the two M714 residues is represented by the black line. (**C**) Top view of S1–S4 and S6 domains. Subunit domains follow the same color scheme as in A. The 6BBJ PDB file was used to produce this figure [70].

The intracellular N-terminal regions contain ankyrin repeat domains (ARD) followed by linkers with two β-strands, β1 and β2, which precede the S1–S4 domains of each subunit and that, together with a β-strand β3 in the C-terminus, give rise to a three-stranded β-sheet that tethers the ARD from the N-terminus to the C-terminus of the same subunit, allowing for interaction with the ARD of another subunit. Following the β-strands, a helix-turn-helix motif and a pre-S1 helix lodge an amphipathic TRP helix in the C-terminus [70]. This TRP helix, a conserved sequence among TRP channels, has been proposed to regulate the gating of these channels and it follows the S6 transmembrane domain in such a fashion that it runs parallel to the membrane and comes nearby to the S4–S5 linker. Thus, it ends up being localized between the cytosolic regions and the transmembrane domains [71,72,73] (Figure 1A).

It must be noted that Arniges and collaborators have shown that the ARDs are important for oligomerization and trafficking of the channel since the absence of these domains results in accumulation of TRPV4 protein in the endoplasmic reticulum [74]. This led these authors to propose a reevaluation of the role of the ARDs in TRPV4 function with respect to previous findings where it was suggested that deletion of these regions still results in functional proteins, albeit less responsive to activation by hypoosmotic stimuli [1]. The ARDs have also been shown to be important for activation of mouse TRPV4 by temperature. In this sense, Watanabe and collaborators have shown that deletion of the first three proximal ARDs results in mutant channels that do not respond to heat [75]; hence, these structures play an important role in temperature detection in this channel. On the other hand, Liedtke et al., showed that in channels where the ARDs have been removed, cell swelling can still activate TRPV4, leading these authors to conclude that the link that the ARDs provide for TRPV4 to the cytoskeleton is not necessary for its response to osmotic stress [22]. Moreover, as will be discussed here, mutations in the ARDs of TRPV4 are associated to genetic diseases such as Charcot-Marie-Tooth disease type 2C [76].

As for the pore of TRPV4, unlike TRPV1 [77], only one constriction was found with the narrowest region (5.3 Å in diameter) being located at residue M714 and it was designated as the lower gate (Figure 1B). Its selectivity filter was shown to be remarkably wide, as compared to other TRP channels, and to coordinate a single hydrated cation, irrespective of the ion valency, at basically the same location [70].

An interesting difference between the TRPV1 and TRPV4 channels is that, in TRPV4 the S1–S4 domain is rotated counterclockwise by approximately 90 °C around the S4 helix, leading to a very characteristic packing interface between S1–S4 and the pore domains. Furthermore, also the way S3 points to the pore and contacts with S6 led the authors to speculate on the possibility of S3 directly interacting with S6 to open the gate [70] (Figure 1C).

We will next explain the function of TRPV4 in the normal physiology of some systems and/or organs that highlight its role as a mechanosensor, osmosensor, and as a chemosensor. It is worth mentioning, that the role of a protein is many times more evidently illustrated when it exhibits a dysfunction that, in turn, often leads to a pathology. Hence, here we have also described some of the diseases that are generated as a consequence of a change in the function of TRPV4.

## 3. TRPV4 and the Vascular Endothelium

Adequate Ca^2+^ signals play a fundamental role in the function of endothelium and in the systems and organs that express endothelial cells, contributing to their homeostasis. Aside from intracellular Ca^2+^ stores that contribute to homeostasis [78], the influx of this ion from the extracellular milieu is extremely relevant for the function of the endothelium and TRPV4 is one of the molecules that participate in this process [27]. Watanabe and collaborators described the activation of TRPV4 in mouse aortic endothelial cells (MAEC), and they corroborated the activation of this channel using a heterologous expression system in HEK293 cells. These authors showed that, in both types of cells, temperatures above 25 °C activated TRPV4 allowing for the influx of Ca^2+^ into the cells and this was reproduced when 4α-phorbol 12,13-didecanoate (4αPDD) was used to activate the channel [53]. Then, the same research group showed that arachidonic acid and anandamide activated TRPV4 in endothelial cells indirectly via cytochrome P450 [68]. Further work carried out by Vriens and colleagues confirmed these results since the block of phospholipase A2 and cytochrome P450 resulted in the inhibition of TRPV4 when activated by osmotic swelling, while activation by temperatures above 25 °C or by 4αPDD remained unchanged [79]. These studies suggested that activation of TRPV4 in vascular endothelial cells contributes to relaxant effects of endocannabinoids on the vascular tone.

Later, by using a combination of techniques including patch-clamp and [Ca^2+^]_i_ measurements, it was shown that TRPV4 was activated in MAEC in response to heat, swelling, 4αPDD, and arachidonic acid, but this activation did not occur in MAEC harvested from *trpv4^−/−^* mice [80].

This prompted several other research groups to broaden the study of the functions of TRPV4 in endothelial cells since it could be of importance for diseases such as hypertension, atherosclerosis, diabetic vasculopathy, vascular tumors, stroke, etc.

It was then shown that the loss-of-function of TRPV4 in endothelial cells of the carotid artery affected the process of vasodilation due to the lack of activation of the channel in the presence of mechanical stimuli (or shear stress), leading to problems in the regulation of the vascular tone, blood pressure, and ultimately affecting the supply of glucose and oxygen to the organs [81].

More recently, the technique of total internal reflection fluorescence microscopy (TIRFM) was used to study microvascular human endothelial cells and it was shown that TRPV4 is present at the plasma membranes of these cells. Moreover, the same authors proposed that GSK1016790A acted by recruiting and activating TRPV4 channels that had previously remained inactive and not by increasing their basal activity [82].

It has been demonstrated that TRPV4 channels give rise to Ca^2+^ transients in the plasma membranes of endothelial cells that lead to the recruitment of other downstream effectors that result in a magnified Ca^2+^ signaling [83]. McFarland and collaborators used an endothelium-specific *trpv4^−/−^* mouse model (ecTRPV4^−/−^) where the channel was specifically knocked down in keratinocytes, to newly show that the artery endothelium from the intact arterial intima of these animals produces larger Ca^2+^ events (via release from internal stores), as compared to the wild-type counterparts but these mutant mice also display deficiencies in “small events”, meaning that TRPV4 channels generate focal Ca^2+^ transients and are essential for vascular homeostasis [84].

Another important aspect of the roles of TRPV4 in the organism are the effects of the influx of Ca^2+^ through this channel at the tight junctions among vascular endothelial cells. There are two important mediators of endothelium-derived hyperpolarization (EDH(F)-mediated) relaxation: TRPV4 and connexins or gap junctions. Both these proteins control vascular tone in concert with nitric oxide (NO) that can mediate relaxation. It has been shown that when ischemia and reperfusion are emulated in arteries from in vivo preconditioned mice that were exposed to repeated cycles of hypoxia-reoxygenation, NO-mediated relaxation is decreased but EDH(F)-mediated relaxation is increased in superior mesenteric arteries [85]; hence, TRPV4 channels and connexins might be important for maintaining vasorelaxation under hypoxia.

Rath et al., also found that preconditioning can preserve vascular function through a mechanism that restores relaxation induced by NO and improves the EDH(F)-mediated response. Furthermore, by using cultured endothelial cells and mice aortas, they found that TRPV4 channels (whose activity and expression levels are increased during hypoxia) and connexins Cx40 and Cx43, help protect the vasculature under an experimental scenario where protection to the vessels is achieved by hypoxic preconditioning and they further strengthen the signaling pathway that depends on NO for these effects to be achieved [85].

Mendoza and collaborators investigated the role of TRPV4 in mouse mesenteric arteries, which maintain a low resistance to changes in blood flow or mechanical stimuli. First they showed that TRPV4 was present in this type of endothelial cells and then they activated the channel with GSK1016790A that allowed for an increase in [Ca^2+^]_i_ and demonstrated that it acted as a second messenger that induced relaxation of the arteries of wild-type mice but not *trpv4^−/−^* mice, concluding that TRPV4 may act as one of the mechanosensitive channels responsible of transducing shear stimuli into Ca^2+^ signaling in vascular endothelial cells [86].

**Figure 2:**
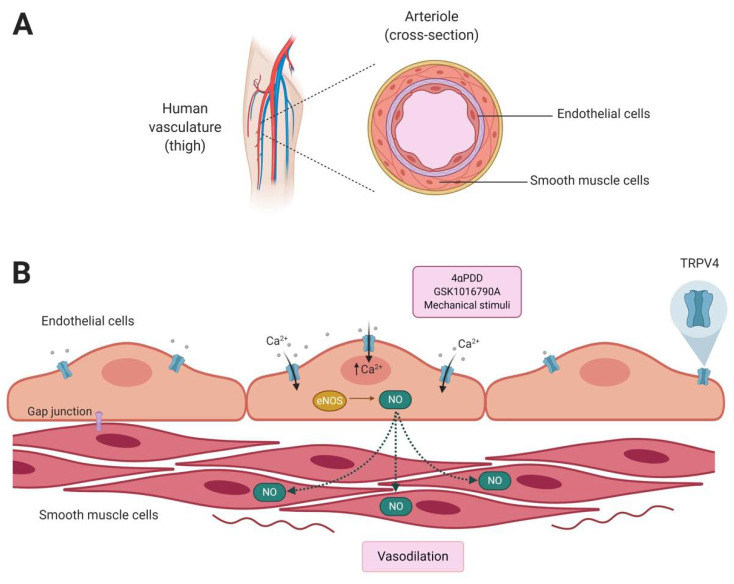
Function of TRPV4 in the vasculature. (**A**) Representation of a region of human vasculature and a cross section of an arteriole. (**B**) Activation of TRPV4 by different stimuli (i.e., 4α-phorbol 12,13-didecanoate or 4αPDD) leads to changes in intracellular Ca^2+^ levels, promoting the activity of the enzyme nitric oxide synthase (eNOS) and then increasing the levels of nitric oxide (NO). NO can cross endothelial cell membranes and activate other signaling pathways in smooth muscle cells, inducing vasodilation. Created with Biorender.com.

The fundamental roles of TRPV4 in the vascular endothelium are consistent with the role of this channel in cardiovascular diseases. For example, it is known that vascular hardening (i.e., atherosclerosis) is associated to the outcome of a cardiovascular (CVD) disease. It has been proposed that activation of TRPV4 can limit vascular inflammation and atherosclerosis [87]. Thus, it follows that endothelial dysfunction in which reduced vasodilation and increased vasoconstriction occur, not only leads to changes in vascular tone but it accelerates the progression of CVDs. Until most recently, the mechanisms by which stiffening of the vascular tone happens had not been clarified. However, Song and collaborators performed experiments using human umbilical vein endothelial cells, and by increasing substrate stiffness, they showed that expression and activity of TRPV4 was reduced, an effect that was followed by an increase in expression of the potent vasoconstrictor endothelin 1 (ET-1) and a decrease in endothelial nitric oxide synthase (eNOS) expression [88] (Figure 2). These authors also identified a stiffness-sensitive microRNA (miR-6740-5p), whose levels were also decreased in stiff substrates. In summary, Song et al., proposed that decreasing the activity of TRPV4 leads to an increase in ET-1 by inhibiting miR-6740-5p and this, in turn, is associated to vascular stiffening that worsens the prognosis of CVD patients [88].

TRPV4 is also expressed in the endothelial microvasculature of the retina and has been associated to diabetic retinopathy. In bovine tissue it was shown that TRPV4 is expressed in this system and when cells were cultured under hyperglycemic conditions, a decrease in the expression of TRPV4 was observed, as compared to control cultures [89].

It was later demonstrated that activation of TRPV4 with GSK1016790A in vitro reversibly increases the permeability of human retinal microvascular endothelial cell monolayers and in vivo it was shown that GSK1016790A augmented permeability of retinal blood vessels in wild-type mice but not in *trpv4^−/−^* mice. Therefore, TRPV4 seems to be pivotal for Ca^2+^ homeostasis and barrier function in retinal capillaries and probably contributes to inner versus outer blood-retinal barrier function (BRB) [24].

Retinal diseases such as diabetic retinopathy exhibit a phenomenon in which there is a breakdown of the blood-retinal barrier that, in turn, leads to neuronal tissue damage and vasogenic edema and then loss of vision [90]. In 2017, Arredondo et al., showed that TRPV4 is expressed in the endothelium and retinal pigment epithelium (RPE) of the BRB and that the use of TRPV4 antagonists could resolve breakdown of the BRB in diabetic rats. They also studied human RPE cell monolayers and endothelial cell systems and found that vasoinhibins (that are endogenous regulators of angiogenesis and vascular function and which have been shown to exhibit lower circulating concentrations in diabetic patients) can block TRPV4, concluding that inhibition of the channel can contribute to conserve the integrity of the BRB and endothelial permeability [91].

Later it was also shown that TRPV4 regulates the migration and tube formation of human retinal capillary endothelial cells, again highlighting TRPV4 as a likely therapeutic target in retinal vascular diseases [92]. In fact, TRPV4 has also been pinpointed as an important molecular effector in glaucoma since new evidences show that its mechanotransducing roles are important within cells in the retina such as: Müller cells, ganglion cell soma-dendrite, and microglia. A dysfunction of the mechanotransduction mechanisms in these cells precedes damage to the retina due to changes in pressure and the antagonism of these mechanosensing protein (among others) could lead to beneficial effects [93].

In the endothelial microvasculature of the human brain (HBMEC) the expression of TRPV4 was also confirmed and shown to regulate [Ca^2+^]_i,_ and thus proposed to act as a protective molecule during damage to brain vasculature [94]. Nevertheless, the angiogenesis that TRPV4 drives after ischemic neuronal death has not been finely examined and it has not been clearly determined if activation of this channel can serve as an effector of neurorestorative events.

Recently, it was evaluated if the activation of TRPV4 could aid functional recovery in rats subjected to transient brain ischemia. By using 4αPDD that was intravenously injected via the tail veins of these animals, it was shown that the infarct volume was reduced by nearly 50% and this was accompanied by better functional outcomes [95]. In this same study, it was also shown that eNOS expression was increased and the expression of vascular endothelial growth factor A (VEGFA) and its receptor VEGFR2 were also augmented, resulting in amplified microvessel density and enhanced neural stem/progenitor cell proliferation and migration. This suggests that activation of TRPV4 may improve poststroke outcomes.

## 4. TRPV4 in the Respiratory Airways and Lung Function

The respiratory system presents sensory innervation of the trachea, glands of the larynx, smooth muscle, bronchial tree and lungs [96,97]. Changes in breathing patterns, dyspnea, and cough are all due to the activation of sensory afferent nerve impulses that travel through the vagal nerve to the central nervous system [98]. There are afferent nerve endings in the upper airway that shield the lower airway from foreign noxious and/or irritant substances [98]. TRP channels are well known to respond to a wide range of molecules that include those that are potentially harmful to an organism [1,99]. In the respiratory airway, TRP channels induce inflammation, airway constriction, mucus secretion, and sneezing and coughing, all of which constitute a mechanism of defense for the respiratory airways [20,98,100].

The TRPV4 channel is expressed in the smooth muscle, fibroblasts, macrophages, submucosal glands, vascular endothelial cells, and in tracheal, bronchial, and alveolar epithelia [20,22,34,101,102,103]. Aside from maintaining the homeostasis of osmotic pressure in these tissues, TRPV4 also integrates stimuli of a diverse nature that translate into Ca^2+^ signals, promoting different responses in the tissues of the respiratory system [104].

Genetical, molecular, and physical regulators come together to ensure adequate pulmonary development. The TRPV4 channel is expressed in apical and basal surfaces of the respiratory airway epithelium, in the subepithelial mesenchyme and in the main pulmonary vessels, all of which are essential for the generation of the dynamic mechanical forces during pulmonary development [30]. Although there is no detailed pathway yet described, TRPV4 has been portrayed as a positive regulator of pulmonary development in the embryonic phase and seems to be important for pulmonary growth based on the mechanism of smooth muscle-contraction [105].

In a murine model of ex vivo embryonic lung explants from mice it was shown that TRPV4 is a modulator of the morphogenesis of the respiratory airway. The activation of the channel promotes the ramification of the respiratory airways, regulates contractility of the smooth muscle favoring its differentiation and augmenting the density of vascularization that supports the growth of the lung in general [30].

In fully-developed animals, Pankey and collaborators studied the response of TRPV4 to the synthetic agonist GSK1016790A in the vascular system of the lungs of rats. They found that when the agonist was injected at low doses it produced a decrease in the pulmonary arterial pressure. Their results showed that GSK1016790A had vasodilation activity in the vascular and pulmonary systems of rats, implicating TRPV4 as a molecular effector of this response [106].

Another research group showed the presence of TRPV4 in the cells of the pulmonary endothelial microvasculature of the mouse (MLMVEC) and of the human (HLMVEC) by studying the effects of oxygen reactive species on [Ca^2+^]_i_ due to TRPV4 activity. In this particular case, it was revealed that H_2_O_2_ activates TRPV4 through a mechanism that requires the presence of a Fyn kinase and, it was concluded that the H_2_O_2_ induces an increase in [Ca^2+^]_i_ as a result of a decrease in transmembrane electrical resistance in microvascular endothelial cells and that agonism of TRPV4 and reactive oxygen species worsen barrier function [107,108].

Ke and collaborators also performed experiments that showed the participation of TRPV4 in pulmonary vasculature. These authors used the myographic technique and GSK1016790A to activate the TRPV4 channel and studied regulation of the vascular tone in arterial rings from the main pulmonary arteries. Their results showed that GSK1016790A relaxed the main pulmonary artery and augmented vascular resistance (vasoconstriction) of pulmonary circulation in isolated perfused lungs, effects that were inhibited by the TRPV4 antagonist AB159908 [109]. These experiments led Ke et al., to propose a mechanism of action of TRPV4 in lung vasculature where the entry of Ca^2+^ through the channel into endothelial cells results in the activation of the intermediate conductance potassium channel (IKCa) and the small conductance potassium channel (SKCa) that leads to the contraction of the pulmonary vascular bed. Conversely, the activation of NO-dependent signaling pathways primes the relaxation (vasodilation) of the main pulmonary arteries [109], suggesting opposing effects for TRPV4 in the lung vasculature.

Interestingly, it was found that there is a close interplay between three molecules that are thought to be pivotal for airway sensory nerve reflexes: TRPV4, ATP (adenosine triphosphate), and P2X3R (P2X purinergic receptor 3, which is activated by ATP) [110]. The use of agonists for TRPV4 and of hypoosmotic solutions led to depolarization of the vagal nerves in humans, mice, and guinea pigs, while antagonists of TRPV4 resulted in a decrease in cough [110]. Hence, it was proposed that TRPV4 is a molecular effector of airway protection and it comes as no surprise that changes in the function of this protein result in respiratory diseases.

Furthermore, Gu and collaborators performed experiments directed at determining the role of the TRPV4 channel in the regulation of breathing in rats. The activation of TRPV4 with GSK1016790A in anesthetized rats induced rapid superficial breathing and potentiated the effects of apnea induced by capsaicin (i.e., chemoreflex) through the activation of TRPV1 in sensory neurons. By using a selective antagonist of TRPV4 (GSK2193874) and by dissecting vagal nerves, these effects were prevented and an indirect role for TRPV4 was suggested in breathing [111]. However, immunocytochemistry and electrophysiological experiments showed that the channel is not functionally-expressed in fibers from sensory neurons, although it is present in macrophages, epithelial and endothelial cells and these authors concluded that regulation of ventilation is due to indirect activation of bronchopulmonary sensory neurons through stimulation of other cells that express TRPV4 in the respiratory system [111].

The endothelial tissue functions as a semipermeable barrier between blood and subjacent tissue that plays a fundamental role in the movement of gases, fluids and molecules of different natures among compartments and maintains cellular homeostasis.

It has been proposed by Parker and collaborators that ion channels activated by cell-stretching were responsible of the loss of vascular permeability in the face of increases in pressure [100] and later TRPV4 was identified as the architect of the loss of endothelial permeability [112] and as promoter of the entrance of Ca^2+^ into cells that leads to the reorganization of the cytoskeleton and to the loss of interendothelial junctions [113].

The activation of TRPV4 with 4αPDD and 14,15-EET induces tearing of the septal endothelium although it maintains the integrity of the interendothelial junctions in the lung of rats and mice, while thapsigargin (an ATPase-mediated inhibitor of the transport of Ca^2+^ into the endoplasmic reticulum) promotes the generation of holes in the junctions of endothelial cells in the extra-alveolar vessels. These observations have prompted the conclusion that damage to extra-alveolar vessels entails different functional consequences to the interruption of the alveolar basal barrier. This, in turn, results in the accumulation of liquid in the alveoli, disallowing for the exchange of gases in isolated lungs from mice and rats, a characteristic symptom of acute lung injury (ALI) [101]. Nonetheless, it was later reported that activation of TRPV4 in wild-type mice increased the permeability in lung endothelial cells without affecting adhesion proteins (i.e., selectins) and without affecting the lung’s wall integrity, a phenomenon that was not observed in *trpv4^–/–^* mice [114].

**Figure 3:**
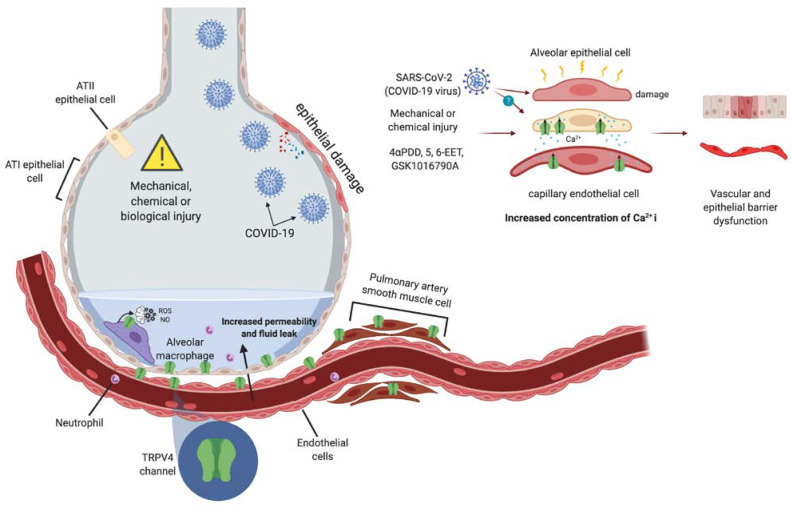
TRPV4 and lung damage. Stimuli such as the exposure to chemicals or mechanical stress (induced by liquids or a ventilator), lead to failure in the function of the alveolo-capillary (endothelial and epithelial cells) barrier with a consequent increase in their permeability, allowing for build-up of liquids in the alveoli. In addition to this, activation of TRPV4 in the alveolar macrophages results in an increase in intracellular Ca^2+^ and in the production of superoxide and nitric oxide (NO). Recently, the SARS-CoV-2 (severe acute respiratory syndrome coronavirus 2) was identified and shown to cause the COVID-19 (coronavirus disease 19) that affects epithelial cells (type I and II) of the alveoli, promoting edema. TRPV4 could be involved in the inflammatory response caused by the SARS-CoV-2 virus. Since TRPV4 overactivation or overexpression can lead to damage in the alveolo-capillary barrier, it has been proposed that inhibitors of TRPV4 could result in a better outcome for COVID-19 patients [147], alveolar epithelial type I and II (ATI and ATII), 4α-phorbol 12,13-didecanoate (4αPDD) and 5, 6-epoxyeicosatrienoic acid (5, 6-EET). ROS, reactive oxygen species. Created with Biorender.com.

ALI and the acute respiratory distress syndrome (ARDS) are characterized by an acute increase in the permeability of pulmonary vascular and endothelial barriers [115]. In animal models of ventilator induced pulmonary injury [116], by liquids [31] and by exposure to chemical compounds (hydrochloric acid or chloride vapors) [117], it was demonstrated that TRPV4 plays a crucial role in the injury to the lungs (Figure 3).

The use of inhibitors of TRPV4 after the induction of ALI was achieved, results in reduced hyperreactivity of the airway pathways, prevents edema, and produces a decrease in arterial pressure. Moreover, it has also been shown that there is a reduction in the pulmonary elastance (elastic deformation by a stressful event that is implicated in alveolar damage) and in the tensoactive-related leakage of proteins, in the infiltration of neutrophils and macrophages to the lung and in the production of inflammatory molecules such as cytokines and promoters of phospholipase A2 [31,117].

On the other hand, when pulmonary injury was promoted through the use of a ventilator, the generated heat induced an increase in pulmonary endothelial and epithelial permeability of wild type mice, which was absent in *trpv4^–/–^* mice or when inhibitors of the channel or of cytochrome P450 were used [116]. A later study by this same research group showed the importance of the expression of TRPV4 in alveolar macrophage cells. By activating TRPV4 with 4αPDD increments in [Ca^2+^]_i_, superoxide and NO in cells from wild type animals were observed and the role of TRPV4 in signaling pathways associated to pulmonary function was further supported [112] (Figure 3).

Moreover, it was reported that the activation of TRPV4 and subsequent Ca^2+^ entry promote the expression of matrix metalloproteinases (MMP2 and MMP9) and a reduction in the TIMP1 (principal metallopeptidase inhibitor 1) in the broncho-alveolar lavage fluid (BALF) of the mouse lung, which is not observed in *trpv4^–/–^* mice. Hence, it was suggested that TRPV4 participates in the recruitment of macrophages and neutrophils during lung injury.

Pulmonary epithelium is formed by different cell lineages: while the larynx and the trachea are coated by squamous epithelia, other tissues of the superior region are coated by cylindrical epithelium that secretes mucus or by caliciform cells that play a similar role in both, the superior and inferior respiratory airways. Clara non-ciliated and non-mucous secretory cells line the most distal region of the pulmonary tree, while epithelial cells type 1 and 2 line the alveoli [118]. All of these different cell types are in charge of preserving the structural integrity of the lungs by allowing for gas exchange, ion transport, growth factor secretion, and by protecting the organism against pathogens and contaminating particles [118].

In primary human bronchial epithelial cells (NHBE) and in the intact trachea of mice, activation of TRPV4 and low-voltage-activated (LVA) calcium channels was reported in response to gentle shearing, a process that allows for a better function of the epithelial barrier since the entrance of Ca^2+^ results in the reorganization of actin and in the formation of stress fibers. Notably, these effects are hindered when there is no Ca^2+^ in the media or in the presence of blockers and/or antagonists of LVA calcium channels or of TRP channels. Hence, it was concluded that shear forces regulate barrier function and the role of these proteins, as well as that of aquaporin 5, was highlighted. Specifically, it was proposed that the entry of Ca^2+^ followed activation of TRPV4 and voltage-gated calcium channels, resulting in the block of solute permeability [119].

As mentioned above, a strong mechanical force such as the one applied by a ventilator to induce pulmonary injury (VILI) will hamper lung function. Excessive mechanical stretch applied in vitro (i.e., using NCI-H292 human pulmonary epithelial cells) or in vivo (murine ventilation model), promotes distension and the consequent opening of the TRPV4 channel and the entrance of Ca^2+^ into the epithelial cells of the lung (Figure 3). This, in turn, leads to the release of cytokines such as interleukins -6, -8 and -1α (IL-6, -8 y -1α). Under this scenario, it was shown that the exposure to the selective TRPV4 antagonist, GSK2193874, reduced Ca^2+^ concentration, and levels of proinflammatory molecules only by about 30%, so this suggested that other mechanically-gated ion channels were involved. Nonetheless, the data demonstrated that TRPV4 also participates in this type of stress induced by stretch and plays a role in the permeability of the pulmonary barrier [25].

Nonetheless, it has also been shown that lipopolysaccharides (LPS) from gram-negative bacteria [120] activate TRPV4 from airway epithelial cells occur independently of Toll-like receptor 4 (TLR4, a protein whose activation leads to the production of inflammatory cytokines) [121], unlike what had been suggested by other groups [122]. Alpizar et al., showed that LPS produce increases in [Ca^2+^]_i_ in mouse and human airway epithelial cells after a few seconds of their application and lead to an increase in CBF and of NO (that has direct antimicrobial and bronchodilation actions). Accordingly, when TRPV4 was pharmacologically inhibited or genetically deleted, augmented ventilatory and inflammatory responses were observed when mice were defied with LPS. In conclusion. TRPV4 is a central molecule in the defenses against bacterial endotoxins [121].

Ciliated cells are in charge of allowing for the propulsion of the mucus gel layer in the respiratory airways and providing for a first line of defense against inhaled particles and pathogens [123]. It has also been demonstrated that TRPV4 is expressed in the ciliated cells of mice trachea and that Ca^2+^ entry through this channel influences the frequency of the ciliary beat (CBF). By using 4αPDD and activating TRPV4, the CBF was increased and this effect was not observed in *trpv4^–/–^* mice. Similarly, it was also described that at 30 or 40 °C and under conditions of high viscosity where TRPV4 was activated, these Ca^2+^ signals also influenced CBF [42].

Interestingly, TRPV4 has also been linked in the response to contaminating particles, such as the diesel exhaust particles (DEP). Although these particles do not seem to directly activate the channel, they promote the activity of proteins, as briefly summarized next: the organic fraction of DEP activates proteinase 2 (PAR-2) and this, in turn, activates TRPV4 (in the moving cilia of the bronchial epithelia) through phospholipase Cβ3 (PLCβ3) and phosphatidylinositol 3-kinase (PI3K) allowing for the entrance of Ca^2+^, which is pivotal for the expression of matrix metalloproteinase 1 (MMP-1), an important molecule for tissue remodeling during development, migration of inflammatory and malignant cells, and diseases of the respiratory airways [124].

Silica nanoparticles (SiNP) are extensively used in cosmetics, food, biotechnology, medical, pharmaceuticals, and chemical industries. These SiNP have been shown to affect the function of the respiratory airways [125]. In this sense, Sanchez and collaborators evaluated the effects of SiNP on the activation of TRPV4 channels from cultured human airway epithelial cells 16HBE and primary cultured mouse tracheobronchial epithelial cells [125]. The authors found that SiNP inhibit the entrance of Ca^2+^ through GSK1016790A-activated TRPV4 channels in both cell types. Moreover, these authors also showed that TRPV4-current inhibition by SiNP (which was dose-dependent) occurred regardless of whether the channels were activated with GSK1016790A or 4αPDD and estimated that nanoparticles rapidly exerted their effects on the channel. In contrast, SiNP produced potentiation of TRPV1 currents in response to capsaicin, leading the authors to conclude that the antagonistic effects of these molecules on TRPV4 were specific [125]. Finally, it was also shown that SiNP decreased the effects of GSK1016790A on ciliary beat frequency. Since SiNP are approximately the same size as the whole TRPV4 channel protein (and double the size of the length of the transmembrane segments of TRPV4), the authors concluded that it is unlikely that these molecules bind to the channel but they might rather produce mechanical disturbances in the plasma membrane that lead to inhibition of TRPV4 [125].

Li et al., went further into determining the steps by which the above described phenomenon takes place. By using human bronchial epithelial cells (BEAS-2B) and human bronchial epithelium (HBE), they activated TRPV4 with 4αPDD and hypotonicity and observed that MMP-1 secretion was increased, while secretion was inhibited with an antagonist of TRPV4 [124]. Moreover, it was shown that the pathological activation of MMP-1 by the enhanced entrance of Ca^2+^ through a mutant TRPV4 with a gain-of-function mutation (TRPV4-P19S) [124] present in a population of humans, could play an important role in chronic obstructive pulmonary disease exhibited by individuals with this genetic polymorphism [126].

A year after this, an artificial lung model was recreated using coats of endothelial and epithelial cells with an alveolo-capillary interphase (a compartment with air and liquid, respectively) and was shown to be capable of imitating the movement of breathing in a normal sate [127]. It was demonstrated that interleukin-2 (IL-2) induces the infiltration of the alveolar canal, that is, an edema due to mechanical tension induced by this molecule. Nonetheless, activation of TRPV4 impedes the leakage, again providing evidence in support of the role of this channel in the face of mechanical forces [127].

In in vitro experiments (using NCI-H292 cells), activation of TRPV4 with various agonists (4αPDD, GSK1016790A, and 5,6-EET) promoted a dose- and time-dependent release of proinflammatory molecules (cytokines, chemokines, etc.) such as interleukin-8 (IL-8) and prostaglandin E2_2_ (PGE_2_). In vivo experiments, after 24 h of intranasal delivery of 4αPDD, showed that it was possible to detect an increase in keratinocyte chemoattract (KC) and in PGE_2_, as well as an infiltration of neutrophils [128]. These results suggest that TRPV4 may be a target for treatment of diseases such as cystic fibrosis, as will be detailed below.

Preservation of the fluid compartment localized at the apical surface of epithelia is pivotal for the function of several organs. In the respiratory airways, the surface liquid layer is important for mucociliary clearance. An adequate balance in the secretion of Cl^−^, HCO_3_ and other anions is crucial for normal physiology of epithelial apical membranes since the secretion of Cl^−^ determines an electrical driving force for the secretion of sodium in the trans-epithelia and, as a result, correct osmotic driving forces for water and secretion products are established [129].

Both production of luminal fluid and its secreted volume are hindered in cystic fibrosis, resulting in the pathophysiological phenomena that typify this disease. This disorder was shown to be mainly caused by mutations in the cystic fibrosis transmembrane regulator protein (CFTR, that is regulated by cyclic adenosine monophosphate or cAMP), which is characterized by severe chronic pulmonary inflammation [130]. Nonetheless, other molecular effectors have been assayed for their importance in the generation or progression of this disease, as are the examples of the transmembrane member 16A (TMEM16A, an anion channel that is activated by Ca^2+^) [131] and of the TRPV4 channel.

It has also been demonstrated that, in vitro and in vivo, TRPV4 is constitutively a part of the signaling pathways of cytosolic phospholipase A_2_ (cPLA_2α_), MAP-kinases, and NF-κB, supporting its involvement in the inflammatory response and potentially in cystic fibrosis pathogenesis [128].

Experiments showed that, in the absence or presence of CFTR, 4αPDD, or GSK1016790A caused a higher degree of mobilization of Ca^2+^, secretion of inflammatory components, KC release, and recruiting of neutrophils [128]. Hence, TRPV4 plays extremely important roles in the airways and is involved directly or indirectly in its pathological states.

Since Cl^−^ channels are fundamental for mucociliary clearance [132], Genovese and collaborators recently tested whether TMEM16A’s activity could be targeted to regulate Cl^−^ transport in the airway epithelia. In their experiments, these authors used the *N*-(2-methoxyethyl)-*N*-(4-phenyl-2-thiazolyl)-2,3,4-trimethoxybenzeneacetamide (Eact) small molecule to directly activate TMEM16A and found that it displayed a mild effect on Cl^−^ transport in airway epithelial cell; however, they discovered that Eact activates TRPV4. Moreover, these observations led this research group to highlight a coupling mechanism between TRPV4 and CFTR-dependent Cl^−^ secretion through the activation of CFTR by the increase in [Ca^2+^]_i_ that results from the activation of TRPV4. The authors also concluded that in non-ciliated cells, Ca^2+^-dependent signaling pathways are elicited by purinergic receptor stimulation leading to TMEM16A (and mucin exocytosis) activation, while in ciliated cells, it is the flux of this ion through TRPV4 the phenomenon that seems to regulate ciliary beat frequency in response to mechanical stress or chemical stimulation. These data imply that the Ca^2+^-dependent signaling events are different depending on the cell type [133].

The smooth muscle is part of the respiratory airways and is present in the trachea and the bronchial tree where it functions as an effector and regulator of the bronchomotor tone, the luminal diameter of the airway and modulates the resistance of the latter. The smooth muscle also plays important roles in embryonic development and in the secretion of cytokines, chemokines, and extracellular matrix proteins [134].

The TRPV4 channel was identified in primary cultures from human smooth muscle cells in the respiratory airway and in the intact tract of guinea pigs, where 4αPDD induced the entrance of Ca^2+^ to the cells and muscle contraction. Interestingly, the activity of other ion channels and signaling pathways was discarded and TRPV4 was pinpointed as essential for the translation of osmotic stimuli in the smooth muscle cells of the respiratory airway [135].

Accordingly, it was also demonstrated that activation of TRPV4 with GSK1016790A promoted the entrance of Ca^2+^ and a strong constriction in samples of ex vivo human bronchia and guinea pig trachea. These effects were abolished using antagonists of TRPV4 and inhibitors of 5-lipoxygenase (an enzyme that synthesizes cysteinyl leukotrienes that are reversible lipophilic constrictors of the respiratory pathway) and of the cysteinyl-leukotriene 1 receptor (cysLT1), suggesting that there is an indirect mechanism by which TRPV4 promotes the production of cysteinyl leukotrienes and muscle contraction [136].

A feature of chronic asthma is the proliferation of airways that contributes to the remodeling and obstruction of these structures and a role for TRPV4 in this process has been suggested [137]. In rats, activation of the channel with GSK1016790A and 11,12-EET produced the entrance of Ca^2+^ to the cells in microdomains termed “Ca^2+^ sparkles”, promoting proliferation of the cells through a signaling cascade that begins with calcineurin that dephosphorylates and promotes the nuclear translocation of NFATc3, which is important for cell proliferation of the smooth muscle under pathological conditions [137].

Finally, it was also reported that KCa3.1 and TRPV4 channels colocalize to human bronchial smooth muscle cells (HBSM) and it was proposed that hyperpolarization of the membrane induced by KCa3.1 leads to an increase in [Ca^2+^]_i_ through TRPV4 and to the subsequent proliferation of asthmatic HBSMs. Furthermore, silencing of any of the genes coding for these ion channels attenuated the entrance of Ca^2+^ and cell proliferation, a process associated to chronic asthma [138].

Another type of cell present in the respiratory airways and the lungs are macrophages that play essential roles in inflammatory processes [139]. Pairet and collaborators had shown, as mentioned above, that equibiaxial stretch in macrophages M1 promotes the release of IL-1α, IL-1β, IL-6, and IL-8, an effect that is prevented when TRPV4’s activity is antagonized with GSK2193874. The physiological implication of this process, according to the authors, is that an increase in the permeability of the endothelia and epithelia of the respiratory airways results in edema and alveolar flooding [25].

Additionally, macrophages have been shown to contribute to the increase in vascular permeability after VILI. Filtration coefficients (K_f_) were measured in the lungs of wild-type mice, of *trpv4^−/−^* and of *trpv4^−/−^* mice inoculated with macrophages from wild-type mice, after which they were exposed to periods of 30 min of ventilation at 9, 25, and 35 cm H_2_O. These experiments showed that wild-type macrophages restored the K_f_ of *trpv4^−/−^* mice to values similar to those of wild type animal lungs [112].

In the same study, it was also demonstrated that activation of TRPV4 with 4αPDD in macrophages promotes an increase in [Ca^2+^]_i_ and of superoxide and NO, which does not occur in *trpv4^−/−^* cells. The authors then suggested that the channel participates in the pathways associated to reactive nitrogen and oxygen signaling, producing a fast increase in the permeability of the lungs after high pressure and volume ventilation. Hence, TRPV4 seems to be a driving force at initiating this type of injury [112].

In infectious processes, roles for TRPV4 have also been demonstrated. For example, it has been shown that this channel constitutes a part of the response mechanism associated to macrophages in the face of pathogens or, as discussed above, of their lipopolysaccharides in murine in vivo and in vitro models [121,140,141].

It has also been recently suggested that TRPV4 can participate in reducing viral infectivity in diseases such as dengue, Hepatitis C, and Zyka [142]. Doñate-Macián and collaborators have shown that TRPV4 can regulate RNA metabolism dependent on DDX3X [142] (a commonly expressed DEAD-box RNA-binding helicase that is sequestered by many RNA viruses [143,144]).

Another viral disease of great importance is COVID-19, which was recently discovered at the end of the year 2019 in China and has been shown to be caused by a beta-coronavirus in assays where human airway epithelial cells were isolated from patients suffering from this disease [145]. The virus was named 2019-nCoV and is now known as the SARS-CoV-2 (Severe Acute Respiratory Syndrome) [145]. Because of its rapid and effective transmission among humans, this disease has been recently (March of 2020) declared a pandemic by the World Health Organization.

People infected with this virus may or may not present clinical symptoms, but when they do these are characterized by high temperature, chest discomfort, and cough, which underlie a pneumonia condition [145].

We know that TRPV4 is activated by cytokines [146]. For example, in an asthma mice model, TRPV4 activation in the membrane promotes an increase in transforming growth factor- beta 1 (TGF-β1) through a signaling pathway involving PI3K and leads to stimulation of the myocardin-related transcription factor A (MRTF-A), which depends on Rho/myocardin. Then, the TRPV4/Rho/MRTF-A pathway activates the expression of fibrosis-related genes that depend on the mitogen-activated protein kinase (MAPK), p38. This, in turn, leads to a higher production of collagen and endothelial fibronectin and to the activation of the inhibitor of the plasminogen activator inhibitor 1 (PAI-1), producing a reduction in the degradation of the matrix [147].

As mentioned in Section 4, both CFTR and TRPV4 play important roles in cystic fibrosis. Specifically, the activation of TRPV4 had been proposed to induce the secretion of pro-inflammatory cytokines/chemokynes from epithelial cells (i.e., PLA2, IL-8, prostaglandin E2, and NF-κB), leading to neutrophil infiltration in response to lipopolysaccharide (LPS) from gram-negative bacteria. Secretion of IL-8 from bronchial epithelial cells in culture, as well as from lungs from intact mice, in response to the activation of TRPV4 was increased when CFTR was inhibited [128].

Finally, in pulmonary injury produced by hydrochloric acid, inflammation of the airways is accompanied by a dramatic increase in pro-inflammatory factors such as keratinocyte-derived chemokine (CXCL1), granulocyte colony-stimulating factor (GCSF), VEGF, IL-1β, IL-6, monocyte chemotactic protein-1, etc. [117,148].

A prominent feature of infection with SARS-CoV-2 it that there is an intense activation of cytokine-driven inflammation cascades [149,150]. Notably, it was recently proposed that TRPV4 may constitute a pharmacological target for patients with COVID-19. It has been proposed that patients in which an inhibitor of TRPV4 is administered (that has already been proven to be safe in a clinical trial) [151], preserve the alveolo-capillary barrier that, in turn, may result in reduced lethality in them (Figure 3).

In summary, the TRPV4 channel plays highly important roles in lung physiology with its activation directly or indirectly influencing the endothelial and epithelial barriers, the smooth muscle and innate immune cell activity.

## 5. Role of TRPV4 in Renal Physiology

TRPV4 is an important molecule for kidney function since, as will be discussed, it regulates the balance of water in cells. Since TRPV4 was described in the year of 2000, it was shown to be abundantly-expressed in the kidney and to respond to hypotonicity [22] and several studies have focused on studying the role of this ion channel in the physiology of the renal system. This channel is absent in the early parts of the kidney tubule (where passive reabsorption of water occurs) but it is present in the distal convoluted tubule and further throughout the kidney. This means that TRPV4 expression is somewhat confined to tubule segments that do not exhibit apical water permeability where transcellular osmotic gradients can develop [152], except for the macula densa region that exhibits apical water permeability and where it is also expressed [47].

By being present in the basolateral membrane of kidney tubular epithelial cells, TRPV4 could respond to changes in osmolarity in the medullary interstitium, tracking local interstitial water balance [152]. In this sense, it has been demonstrated that when the osmolarity decreases in the renal medulla, ATP is released from epithelial cells in the thick ascending limb. When renal tubules are treated with a chemical agonist of TRPV4, ATP is released in the thick ascending limb under isotonic conditions, but if TRPV4 is knocked down then there is a decrease in ATP release under hypotonic conditions [153]. The conclusion is that ATP is released in the thick ascending limb when there is cell swelling that activates TRPV4 (i.e., a reduction in medullar osmolarity) and the channel regulates the osmotic balance by regulating water secretion in the kidney.

Nonetheless, TRPV4 also regulates water secretion through a mechanism that involves the central nervous system. Knockout of TRPV4 in mice results in less water consumption and increased serum osmolarity than wild-type animals. When a hyperosmotic challenge was applied, the levels of vasopressin (AVP) in the plasma were much lower in *trpv4^−/−^* than in wild-type mice, and although these animals exhibited average renal responses to AVP, the expression levels for c-FOS (a marker of neuronal activity) in the hypothalamus (circumventricular organs) were decreased, meaning that osmo-sensation was compromised in the central nervous system [23].

Much about our understanding on how TRPV4 plays important roles in kidney physiology of mammals comes from studies in worms (i.e., *C. elegans*) that express OSM-9 (for which TRPV4 is the mammalian homologue), a protein important for olfactory signaling and hyperosmotic noxious stimuli detection. In this sense, it has been demonstrated that transgenic worms that lack OSM-9 exhibit impaired mechano-sensory and osmo-sensory responses as well as flawed olfactory responses [33].

Before the cloning of this sensor for hypotonic stress and cell swelling, there were considerable efforts to understand the underlying mechanisms for the detection and responses to this type of stimulus [154]. It had been suggested that the response to hypotonicity could be due to activation of a receptor-tyrosine kinase [155,156] and then it was shown that hypotonic stress indeed led to tyrosine phosphorylation of TRPV4 (by a member of the Src family of tyrosine kinases, Lyn), both in a heterologous expression system and in mouse distal convoluted tubule cells in culture [157]. It was also described that residue Y253 was the site in TRPV4 that was phosphorylated by Lyn in response to hypotonic stress and this study constituted the first one to show that a TRP channel could be directly regulated by tyrosine phosphorylation [157].

Other kinases have also been shown to influence the physiology of TRPV4 and these include the family of lysine deficient protein kinases (WNK). WNKs have been shown to influence the expression of important molecular effectors of the kidney such as the thiazide-sensitive Na^+^-Cl^−^ cotransporter and in fact, mutations in members of the WNK family of protein kinases can lead to diseases such as polygenic human hypertension [158]. Accordingly, it has been demonstrated that WNK4 and WNK1 downregulate the function of TRPV4 in HEK293 cells by decreasing its expression in the plasma membrane, although levels of total TRPV4-protein remain unaffected, pinpointing a role for these WNKs in the trafficking of TRPV4 to the surface of the cell [159]. Both TRPV4 and isoforms of WNK are expressed in the distal nephron, suggesting that TRPV4’s function may be regulated by these molecules in a physiologically relevant scenario.

It has also been shown that TRPV4 is activated by cell swelling through mechanisms that involve phospholipase A2-dependent generation of arachidonic acid and polyunsaturated fatty acids or PUFAs (i.e., 5, 6-EET) through the cytochrome P450 epoxygenase-dependent pathway in mammalian cells [68,80]. Nonetheless, studies using yeast-expression systems have shown that indeed TRPV4 participates in mechanotransduction in response to hypotonic conditions but in a PUFA-independent fashion since these cells do not produce these molecules [160].

Since TRPV4 is expressed in regions of the nephrons that are water-impermeant, it was suggested that this channel plays important functions in the detection of osmotic stimuli and that it may regulate blood pressure in the presence of increased salt intake. To test this hypothesis, Gao and collaborators used rats that were fed normal- and high-sodium diets as well as 4αPDD-intravenously. In this study, the authors concluded that the high-salt diet produced an increase in the expression of TRPV4 in DRG and mesenteric arteries but not in the renal cortex and medulla. Moreover, when animals were challenged with a high salt diet and TRPV4’s activity was blocked, the increase in blood pressure was larger than when the channel was not blocked, suggesting that TRPV4 plays a protective function against this process [161].

In general, cilia are considered sensory organelles that can detect physical and chemical stimuli, and in the kidney, renal epithelial cells are subject to changes in fluid flow that can bend them, leading to an increase in [Ca^2+^]_i_ [162]. It has been shown that the protein complex formed between another member of the TRP family of ion channels, TRPP2 (a product of the polycystin kidney disease 2 or *pkd2* gene), and polycistin-1 (a product of the polycystin kidney disease 1 or *pkd1* gene) is important for the entrance of Ca^2+^ under these conditions. If the presence of cilia is eliminated or if these proteins are affected by mutations, a disease called autosomal dominant polycystic kidney disease occurs, and in this disease, the liver, pancreas, and kidney exhibit fluid-filled cysts [163,164]. TRPP2 is channel that lacks mechanosensitivity [165]; nonetheless, it has been shown in vitro that this channel can form heterotetrameric complexes with TRPV4 [166] and that these are thermo- and mechano-sensitive [167].

There seems to be a clear correlation between cyst formation and the requirement of cholangiocyte proliferation and decreased levels of [Ca^2+^]_i_ in these cells, at least in animal models of the autosomal-recessive polycystic kidney disease (ARPKD) [168,169]. Cholangiocytes express the TRPV4 channel and its activation leads to an increase in [Ca^2+^]_i_ [44]; hence, it has been proposed that pharmacological activation of TRPV4 might inhibit the hyperproliferation of cholangiocytes in polycystic kidney rats (PCK). In 2010, it was shown that the expression of TRPV4 in PCK cholangiocytes is increased and that activation of the channel in these cells not only inhibited cell proliferation by 25%–50% and cyst growth by 3-fold in cultures, but also decreased the cystic area in in vivo experiments [170].

**Figure 4:**
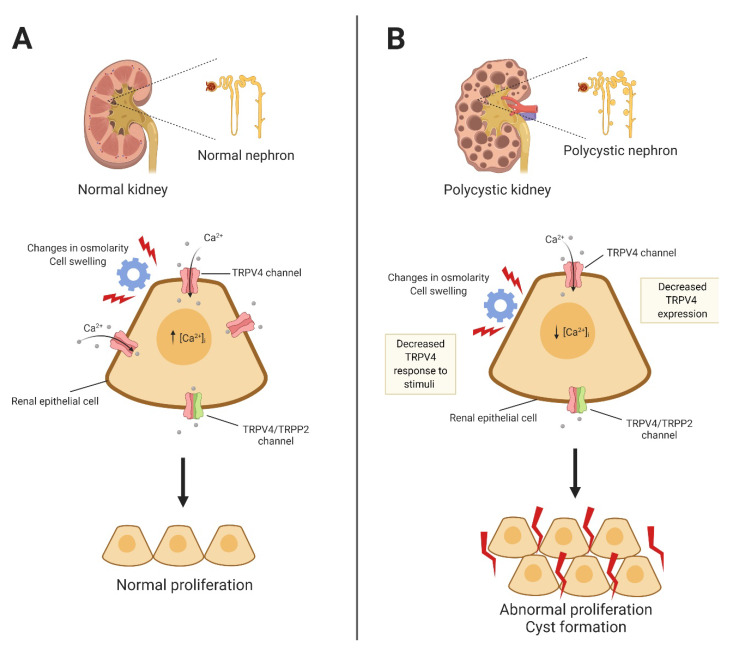
The activity of TRPV4 influences cyst growth and proliferation. (**A**) shows a normal kidney and nephron (top panel). The middle and lower panels show that when TRPV4 is adequately-expressed or activates normally, cell proliferation is kept under control. On the contrary, (**B**) shows that a polycystic kidney is full of fluid-filled structures which is a result of the lack of expression of correct function of TRPV4 (middle panel), resulting in abnormal proliferation and cyst formation (lower panel). Created with Biorender.com.

Moreover, it was shown in isolated collecting duct (CD)-derived cyst monolayers are dilated (which are dilated) that the activity of TRPV4 was impaired, it was abnormally located at subcellular levels and that its glycosylation was disrupted; hence, the levels of [Ca^2+^]_i_ and the signaling pathways associated to this ion were also diminished. Nonetheless, in the nondilated CDs (from normal tissue), the activity of TRPV4 was preserved and so was their mechanosensitivity [171]. In this study it was also shown that the systemic pharmacological activation of TRPV4 for long periods of time progressively reinstated the mechanosensitivity of cyst cells and diminished the progression of ARPKD in the rat model [171] (Figure 4).

Furthermore, it has been proposed that activation and glycosylation of TRPV4 in humans is also decreased and that stimulation of this channel may constitute a possible therapeutic target for the progression of ADPKD in humans [172].

It was mentioned above that TRPP2 and TRPV4 form heteromers in vitro, but a very recent study analyzed these complexes in vivo in cortical collecting ducts from conditional knockout mice without cilia (*Ift88^−/−^*) and with cilia (*Ift88^flox/flox^*) [173]. Using the patch-clamp technique it was shown that apical channel activity in the ducts from *Ift88^flox/flox^* did not correspond to the expected conductances of the TRPP2-TRPV4 heterotetramer, while their counterparts from *Ift88^−/−^* mice primarily showed the conductance expected for this complex (23 pS) [173]. Saigusa et al., also showed that mRNA levels for TRPP2 or PKD2 and TRPV4 were elevated in isolated cortical collecting ducts when cilia were lost [173]. In conclusion, unregulated increases in Ca^2+^ flux occur as a consequence of the loss of cilia and this may contribute to the generation or progression of PKD.

Other studies have shown that changes in the expression of TRPV4 in renal tissue may also lead to problems in the ability to regulate cell volume when there is a diabetic background. Hills et al., demonstrated that in human collecting duct (HCD) cells exposed to high glucose concentrations, the expression of TRPV4 was reduced by 54% and suggested that this may produce a decline in the capacity of the collecting duct to regulate volume decreases, leading to electrolyte imbalances such as the ones that are observed un diabetic nephropathies [174].

A malfunction of TRPV4 has also been implicated in damage to the kidney after acute renal ischemic reperfusion injury (IRI), characterized by endothelial dysfunction and tubular injury. Using a model of induced IRI (the left renal pedicle was clipped after a right-sided nephrectomy) in wild-type and *trpv4^−/−^* mice, it was shown that serum creatinine levels, that reflect damage to the kidneys, were higher in *trpv4^−/−^*, as compared to the wild-type animals [175]. Thus, the results of this study suggested that TRPV4 could play a protective role against IRI.

In contrast, another study performed in neonate pigs showed that block of TRPV4 alleviates IR-induced renal insufficiency in these animals through a mechanism that seems to involve angiotensin II signaling that promotes inflammation, vascular dysregulation, and fibrosis in acute kidney injury [176].

## 6. The Musculoskeletal System and TRPV4

Musculoskeletal tissues have been shown to be mechanosensitive, as exemplified by the remodeling of the bone as a physiological response to mechanical loading (during physical activity) [177], a phenomenon for which the molecular effectors remained unclarified for a long time. The renewal of osteoblasts that form the bone from mesenchymal stem cells (MSCs) is pivotal for skeletal homeostasis. In the recent years, growing evidences have implicated TRPV4 in this physiological process [178,179]. For example, it has been shown that TRPV4 is expressed in MSCs, especially in areas of elevated strain in the primary cilium and that this channel is required for mechanotransduction that, in turn, is necessary for Ca^2+^ signaling induced by oscillatory fluid shear [180]. Moreover, in 2020 it was demonstrated that TRPV4 and the Piezo 1 and Piezo 2 channels influence stretch-dependent Ca^2+^ responses in chondrocytes. To show this, Du and collaborators transfected cultured primary chondrocytes in which these channels were knocked down by using siRNAs and measured stretch-evoked [Ca^2+^]_i_ influx [181]. Their results show that all of these channels are required for Ca^2+^ signaling in response to stretch. Nonetheless, these authors were also able to discern that TRPV4 performed a key role in chondrocyte responses to physiological levels of strain. and conversely, Piezo 1 and 2 were important to attain Ca^2+^-dependent signaling pathways in response to levels of strain that could produce injuries.

**Figure 5:**
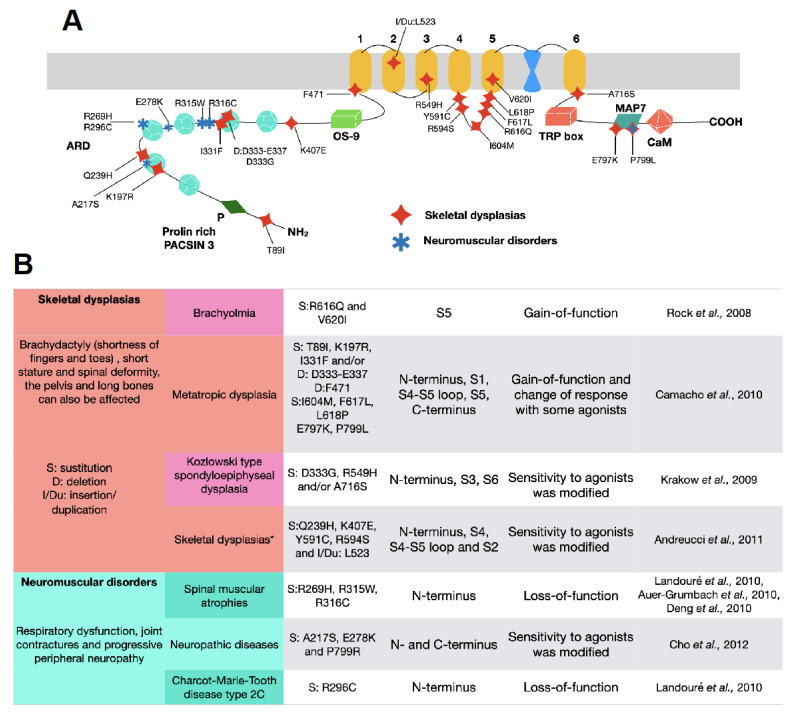
Location of mutations in different regions of the TRPV4 structure that produce skeletal dysplasia and neuromuscular disorders. (**A**) The top panel represents a subunit of the TRPV4 channel. I/Du = Insertion/Duplication. (**B**) The bottom panel summarizes the data in the top panel and the type of disease they produce as well as the effect of the mutation in channel function. PACSIN 3, protein kinase C and casein kinase substrate in neurons protein 3; ARD, ankyrin repeat domain; OS-9, osteosarcoma 9; TRP box, Transient Receptor Potential box; MAP7, microtubule associated protein 7; CaM, calcium calmodulin.

Certain diseases were previously thought to be distinct clinical phenotypes until it was discovered that there was a common underlying molecular basis: their association with the mutations and malfunction of TRPV4 (Figure 5). Presently, these disorders have been grouped into skeletal dysplasias (metatropic dysplasia, parastremmatic dysplasia, Maroteaux type spondyloepiphyseal dysplasia, Kozlowski type spondyloepiphyseal dysplasia (SMDK), autosomal dominant brachyolmia, familial digital arthropathy-brachydactyly), and into neuromuscular disorders (congenital distal spinal muscular atrophy, scapuloperoneal spinal muscular atrophy, Charcot-Marie-Tooth disease type 2C), which vary in severity. Skeletal dysplasias exhibit brachydactyly (shortness of fingers and toes), and depending on the severity of the disease, there is also short stature and spinal deformity, the pelvis, and long bones, which can also be affected, and sometimes the life span of the individuals is reduced. On the other hand, neuromuscular disorders present themselves with respiratory dysfunction, joint contractures, and progressive peripheral neuropathy [182].

All of these diseases, which are grouped into two large categories (i.e., neuromuscular disease and skeletal dysplasia), encompass progressive degeneration of peripheral nerves or lack of establishment and development of the hard-skeletal tissues.

It had been previously shown that inactivation missense mutations in the PkdI (polycystic kidney disease) gene that encodes the polycystin-1 (PC1) membrane protein led to tardy intramembranous and endochondral bone formation in a mutant mice (Pkd1^mlBei^) strain [183]. A link between this discovery and the role of TRPV4 in the skeletal system was later made since it had been shown that PC1 activates TRPV4 through a G-protein coupled receptor (GPCR) mechanism [184].

In 2008, a study described that gain-of-function mutations in TRPV4 produced autosomal dominant brachyolmia [185], a disease characterized by individuals with short trunk, scoliosis, and short stature [186]. The authors of this study described point mutations in the TRPV4 channel in two families that presented the brachyolmia phenotype, R616Q and V620I. By producing these mutations in the channel, they performed in vitro experiments and electrophysiological recordings and showed that both of these mutations gave rise to a TRPV4 channel with amplified constitutive activity and higher channel activation in response to 4αPDD and to mechanical stimulation [185].

Later, another study was published in which it was described that the TRPV4 channel controls the levels of Ca^2+^ flowing into large osteoclasts and increases bone mass by impairing bone resorption in the *trpv4^−/−^* mouse [29]. This study in particular assessed the identity of TRPV4 as a molecule responsible of allowing the entrance of Ca^2+^ [29] that permits for osteoclast differentiation through a mechanism that involves receptor activation of NF-κB ligand (RANKL). This, in turn, leads to gene transcription of nuclear factor-activated T cells 1 (NFATc1) that translocates to the nuclei of osteoclasts and results in specific gene transcription and differentiation of these cells [187].

Other studies have shown that other point mutations in TRPV4 also result in other types of dysplasias. For example, the R594H, D333G, and/or A716S substitutions were present in patients with SMDK, which is characterized by severe scoliosis and mild metaphyseal abnormalities in the pelvis [188]. When these mutations were examined in vitro, it was found that the D333G and R594H mutant channels displayed larger constitutive currents in the absence of a TRPV4 agonist. The shape of the current-voltage (IV) curve of the channels remained unchanged but the sensitivity to agonists was modified, since R594H was insensitive to 4αPDD and mildly sensitive to cell-swelling and arachidonic acid. In contrast, the response of D333G to this agonist was increased as compared to the wild-type TRPV4 channel, while the A716S mutation displayed similar constitutive activity to that of the wild-type channel but was also insensitive to 4αPDD, cell-swelling, and arachidonic acid [188].

Metatropic dysplasia is also a clinically heterogeneous skeletal dysplasia where the affected individuals present a short trunk with kyphoscoliosis (abnormal curvature of the spine), short extremities, squared-off jaw, and midface hypoplasia (upper jaw, eye sockets, and cheekbones exhibit a lesser degree of growth than the rest of the face). Camacho and collaborators showed that this disease is dominantly inherited with locus homogeneity by analyzing genetically-independent cases where the patients were in the range of being mildly affected to dying soon after being born (neonatal lethal) [189]. The mutations observed in the 10 patients from this study were: T89I in the beginning of the N terminus; K197R, I331F and/or a D333-E337 deletion insertion in the fifth ARD; F471 deletion in the S1, I604M in the S4–S5 loop, F617L and L618P in the S5, and E797K and P799L in the C terminus. By studying the effects of two of these mutations, I331F and P799L, the authors found that these channels produced larger basal currents with channels being open in the absence of agonists and responses were higher, as compared to wild-type channels, when stimulated with 4αPDD and cell swelling but not with arachidonic acid [189]. Thus, the results were similar to those described above for SMDK [185,188].

Five more TRPV4 mutations were found to be present in patients from 21 families presenting skeletal dysplasias: K407E, R594S, Q239H, Y591C, and the insertion/duplication L523 [190], and it was later suggested that mutations that caused skeletal dysplasias could also cause neuropathic diseases.

Patients with spinal muscular atrophies (SMA) and hereditary and sensory neuropathies (HMSN) constitute other examples of clinically and genetically heterogenous disorders of the peripheral nervous system. These diseases are characterized by the loss of motor neurons and muscle atrophy, including respiratory muscles, and is a leading cause of death in infants (i.e., SMA) and by atypical neural development and degradation of the neural tissue (i.e., HMSN). In these cases, it has also been reported that specific mutations in the *trpv4* gene can cause scapuloperoneal SMA (SPSMA), distal SMA, and HMSN 2C.

Auer-Grumbach and collaborators [191] found that mutations in the ARDs (R269H (also studied by [76]), R315W, R316C [192]) of TRPV4 were present in 10 individuals from one family that displayed mild to severe congenital distal SMA; SPSMA or HMSN2C (or Charcot-Marie-Tooth disease type 2C) phenotypes. The authors constructed channels with these mutations and studied their functional features in a heterologous expression system where they determined that, not only was surface expression decreased and the influx of Ca^2+^ was reduced when these mutant channels were challenged with 4αPDD or hypotonicity [191], but also that the expression of these mutants was highly toxic in cultured neuronal cells (dorsal root ganglia or DRG) [76]. Another mutation of the same residue (R296C) in the ARDs of TRPV4 was also found by Landouré et al., and shown to be associated to HMNSN2C [76].

In a study involving patients from 21 families, five more TRPV4 mutations were found to be associated to skeletal dysplasias: K407E, R594S, Q239H, Y591C, and the insertion/duplication L523 [190], and it was suggested that mutations that caused skeletal dysplasias could also cause neuropathic diseases, leading researchers to describe the presence of another three mutations in 3 patients: E278K, P799R, and A217S [193] (Figure 5).

Disruption of the function of TRPV4 is not only relevant for humans and other mammals, as is exemplified by a recent study where it has been shown that disruption of several genes of worm (*C. elegans*), including OSM-9/TRPV4, leads to changes in their ability to crawl and swim, exhibiting debilitated movement [194].

All of the above evidences exemplify the profound importance of an adequate function of this ion channel in skeletal-muscular physiology.

## 7. Skin and TRPV4

In the skin, TRPV4 participates, along other TRP channels, in preserving skin homeostasis by regulating hair follicle growth, conservation of the skin barrier, immunological (and inflammatory) responses, etc. [195]. Moreover, since TRPV4 is a thermosensitive TRP channel, it functions as a detector of changes in temperature, but also as a sensor of chemical and mechanical stimuli [195].

In mouse keratinocytes, TRPV4 is activated by warm temperatures leading to a faster regeneration of the barrier, an effect that is also observed when specific agonists of the channel are used [196]. TRPV4 has also been found in the cell–cell junctions of the epidermis where, in concert with the E-cadherin complex to which it associates, it supports cell–cell junction development and the generation of a close-fitting barrier amid keratinocytes [195,197,198,199]. The latter has been attributed to a role of TRPV4 in upregulation of occludin and claudin-4, both of them tight junction structural proteins [200].

The TRPV4 channel has recently been described as an important mediator of itch, which is an unpleasant sensation that leads to the generation of the scratch reflex, one that severely impacts the quality of life of the individuals who suffer from it [201]. Clinically, itch is either of a peripheral or a central origin; however, here we will focus on peripheral itch since this is the one that, in many cases, is due to the function of TRP channels [202].

Peripheral itch, also termed pruritoceptive itch, occurs in the skin and has several causes: inflammation, dryness, or exposure to pruritogenic agents [201]. At the cellular level, itch requires the involvement of specific cell mediators that include skin and immune cells and sensory neurons [202]. The interplay between these cells produces the transduction of the pruritogenic signal that travels to the brain where it is interpreted and resolved as a motor response: scratching [202]. Likewise, these cellular players require several other locally-released mediators that activate different receptors such as G protein-coupled receptors (GPCRs) and ion channels like TRP channels that are expressed at the plasma membrane of each type of these cells.

TRP channels have a pivotal role in the physiology and pathology of the skin, and recently the TRPV4 channel has emerged as a pharmaceutical target for skin disorders like rosacea, ichthyosis, psoriasis, and contact and atopic dermatitis [32].

As previously mentioned, TRPV4 is expressed in mice neurons from the central nervous system and discrete expression has also been observed in sensory neurons from the trigeminal ganglion [22]. Nonetheless, it is important to note that a strong signal for TRPV4 mRNA was also detected in Merkel cells, which are highly specialized epidermal cells [203].

Immunohistochemical analysis in mice demonstrated that the TRPV4 protein is expressed in small and large diameter dorsal root ganglion (DRG) neurons and it was also confirmed that TRPV4 is expressed in mechanosensitive Merkel and Meissner cells [203]. Additionally, TRPV4 is expressed in keratinocytes, where it is activated by warm temperatures [41] and where this channel has an essential role in the cell–cell junctions [197].

Since TRPV4 is expressed in the cellular components that generate itch, it is possible that this channel could have an important role in regulating the function of these cells and dysregulation of TRPV4 expression and/or function could contribute to some dermatologic pathologies that are accompanied by an itch sensation.

The first association between TRPV4 and the itch sensation was initially determined in burn scars from patients with post-burn pruritus where an increase in TRPV4 expression was observed. In contrast, burn scars of patients without pruritus showed lower TRPV4 expression than the former group [204]. These observations suggest that TRPV4 overexpression is associated to post-burn pruritus, although the molecular mechanism of this regulation remains unresolved.

The roles of TRPV4 in pathological skin conditions have also been studied in patients with chronic idiopathic pruritus, which display severe skin dryness and chronic itch of an idiopathic nature. The analysis of TRPV4 expression in skin biopsies from these patients revealed that it is upregulated, as compared to samples from healthy patients [205].

Additionally, two mouse skin itch models have strengthened the role of TRPV4 in itch production. These models are the dry skin-associated chronic itch (induced by the injection of an acetone/ether mixture followed by water in the rostral back) and the spontaneous scratching model (associated with squaric acid dibutylester–induced allergic contact dermatitis) [205].

These mice models display TRPV4 overexpression and intense scratch behavior. This response decreases when the mice are treated topically or injected intraperitoneally with a TRPV4 inhibitor [205]. Furthermore, the establishment of these itch models in the *trpv4^−/−^* mice also showed a decrease in scratch behavior [205]. These results support that TRPV4 channels are essential to itch produced in these type of mice models.

Likewise, the relevance of TRPV4 channels in the generation of itch has been revealed by using a TRPV4^GFP^ transgenic mice line, which demonstrated that TRPV4 channels are functionally expressed in keratinocytes and dermal macrophages, as evidenced through the green fluorescent protein (GFP) [205]. TRPV4 expression in keratinocytes and macrophages is required to induce itch in the dry-skin and allergic contact dermatitis mouse models, respectively. Furthermore, both models also require the release of serotonin (5-HT) from platelets that activate specific serotonin receptors in free nerve endings to transduce the itch signal [205].

**Figure 6:**
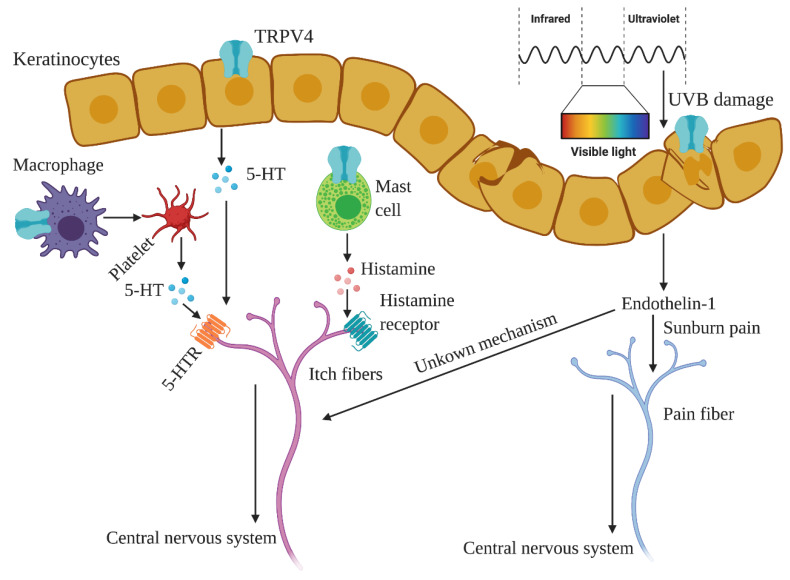
Three itch pathways associated to the function of TRPV4. When there are existing conditions such as dry skin, contact dermatitis, rosacea, etc., activation of TRPV4 in keratinocytes and in macrophages (that activate platelets) leads to the release of serotonin (5-HT) from these cells. 5-HT binds to its receptors in the itch fibers and this signal is transduced as itch in the central nervous system. Something similar occurs when TRPV4 channels in mast cells are overactivated or overexpressed, leading to the release of histamine that also activates receptors in itch fibers. It has also been proposed that UVB light damage, leads to the production of endothelin-1 that causes sunburn pain but could also cause post solar-burn itch through a mechanism not yet understood. 5-HTR, serotonin receptor. Created with Biorender.com.

In support of the existing interplay between TRPV4 and the serotoninergic pathway, previous studies have demonstrated that 5-HT produces acute itch through the activation of TRPV4 [206] (Figure 6). This was determined using an acute itch mouse model, where wild-type animals were intradermally injected with 5-HT in the cheek. These mice displayed scratch behavior, which was attenuated in the *trpv4^−/−^* mice [206]. Moreover, dissociated DRG neurons from wild-type and *trpv4^−/−^* mice were used to carry out fluorometric assays, demonstrating that 5-HT produces Ca^2+^ influx in wild-type DRG neurons, an effect that was not observed in neurons from *trpv4^−/−^* mice [206].

In summary, under certain conditions, keratinocytes and macrophages overexpress TRPV4 channels, stimulating the release of 5-HT from platelets (through an unknown mediator). 5-HT then activates specific receptors (i.e., 5-hydroxytryptamine receptors 2 and 7 (HTR2 and HTR7) ) and/or ion channels such as TRPV4 [205,206].

Similarly, it has been reported that chloroquine can induce scratch behavior in a TRPV4-dependent manner. Specifically, it was determined that injection of chloroquine to wild-type animals displayed scratch behaviors while, in their *trpv4^−/−^* counterparts, this phenomenon was reduced [207]. Interestingly, chloroquine induces itch in a fashion that is dependent on TRPV4 expression in DRG neurons, where the TRPV1 channel is also expressed. Neurons co-expressing these channels showed that TRPV1 is a facilitator of TRPV4 activation since neurons that did not express TRPV1 displayed reduced Ca^2+^ influx in response to specific TRPV4 agonists [207].

Accordingly, mice co-injected with TRPV1 and TRPV4 antagonists showed a decrease in chloroquine induced pruritus [207], indicating that TRPV1 and TRPV4 cooperate in transducing pruritogenic stimuli and in producing the scratch response.

The above results exemplify the roles of TRPV4 in the itch pathway in response to 5-HT and chloroquine. Moreover, it has also been shown that TRPV4 plays an active role in histamine induced pruritus in wild type mice, a response that is diminished in *trpv4^−/−^* mice intradermally injected with this molecule [208]. Moreover, this pruritogenic effect is also decreased in an inducible keratinocyte *trpv4^−/−^* mice line, suggesting that TRPV4 expressed in keratinocytes is essential for the effects of histamine [208] (Figure 6).

Additionally, histaminergic itch produced through TRPV4 requires the activation of downstream signaling pathways that induce the phosphorylation of the extracellular signal-regulated kinase (ERK). The block of this phosphorylation considerably reduces histamine-induced itch [208]. This whole mechanism represents a remarkable interplay between the TRPV4 channel that is expressed in keratinocytes and a pruritogenic agent such as histamine in order to induce itch.

Similar results were obtained using ET-1, another pruritogenic agent that produced scratch behavior in mice [208,209]. ET-1 pruritogenic actions decrease in the *trpv4^−/−^* mice and also in the inducible keratinocyte *trpv4^−/−^* mice model [208]. Like histamine, ET-1 induces Ca^2+^ influx in keratinocytes from wild type mice; however, this influx is decreased in the keratinocytes from *trpv4^−/−^* mice [208].

Remarkably, it has been shown that ET-1 and TRPV4 expression are upregulated in human skin overexposed to UVB radiation [210], although this was mainly associated with sunburn pain, it is possible that it also produces post solar-burn itch (Figure 6).

Furthermore, the TRPV4 channel has also been associated to psoriasis, an itching condition characterized by chronic skin inflammation. By using an induced psoriasis mice model, it was determined that TRPV4 is overexpressed in DRG neurons isolated from these mice [211]. Channel overexpression was observed from the second day of the induction of psoriasis, a period where the alloknesis score (itch evoked by light cutaneous stimuli) significantly increased in these mice [211].

Another feature of psoriasis is the reduction in keratinocyte differentiation and the increase in proliferation of these epidermal cells, producing the accumulation of cells on the epidermis and intense itch. This unbalance can be restored by the action of baicalein, a flavonoid found in the mushroom *Scutellaria baicalensis* [212]. Experiments performed using the HaCat cell line (immortalized human keratinocytes) demonstrated that cells treated with baicalein exhibited a decrease in their proliferation and an increase in the expression of some keratinocyte differentiation markers such as keratin 1 and 10 [212].

Under this scenario, cell differentiation was dependent on TRPV4 activation by baicalein, which produced an increase in Ca^2+^ influx and phosphorylation of ERK, both of which are necessary for an increase in keratinocyte differentiation [212]. These results suggest that TRPV4 activation could be beneficial for restoring the balance between proliferation and differentiation in keratinocytes [212].

Finally, TRPV4 expression has also been linked to rosacea, a chronic inflammatory skin disease where some patients experiment an itchy sensation [213]. As we mentioned before, it has been reported that TRPV4 is expressed in keratinocytes from human healthy skin biopsies; remarkably, the skin biopsies from rosacea-affected skin patients additionally display TRPV4 expression in immune cells while the skin biopsies from healthy humans have lower TRPV4 expression levels in these cells [213]. Furthermore, the upregulation of TRPV4 expression has been determined in the skin of a murine-rosacea model.

The upregulation was specifically observed in mast cells, which have an active role in the inflammation state associated to this dermatological condition [214]. This effect was mimicked in human mast cells (hMC) treated with the antimicrobial proteolytic cathelicidin fragment LL37, which is upregulated in rosacea patients, and it is a ligand of the Mas-related gene X2 (MrgX2) GPCR [214].

The increase observed in TRPV4 mRNA levels was associated with the Gα_i/0_ pathways since hMC co-treated with LL37 and pertussis toxin did not exhibit a rise in TRPV4 expression [214]. This suggests that TRPV4 overexpression in hMC could increase Ca^2+^ influx, producing degranulation of these cells and exacerbating the inflammatory condition. Although rosacea is a chronic inflammatory skin disease, it is not a strictly itching condition because only some patients have pruritus. Nonetheless, expression of TRPV4 in immune cells could contribute to a generalized inflammatory condition and itch produced in some patients [213].

The growing evidence on the involvement of TRPV4 channels in the generation and/or transduction of pruritogenic signals raises the possibility of designing new pharmacological tools that target TRPV4 to avoid itch produced in the main dermatological disorders mentioned. For example, over the last 70 years, crotamiton (N-ethyl-ocrotonotoluidine) has been used as a scabicide and anti-pruritogenic agent [215]. The anti-itch action of this compound was not completely understood; however, recently it was described that this compound inhibits itch produced by a TRPV4 selective agonist [215].

Furthermore, electrophysiological experiments have demonstrated that crotamiton inhibits TRPV4 activation and removal of the chemical during the experiments leads to the appearance of a large conductance state that is accompanied by changes in the reversal potential of this channel [215]. The physiological consequences of these last effects have not been studied but they could contribute to the burning sensation that is present in some cases of patients treated with crotamiton.

To summarize, the above described experimental evidence highlights TRPV4 as a target for itch relief. Although studies are still necessary in order to better understand the molecular roles of TRPV4 in itch, it is clear that this channel influences the interaction among main cellular components necessary for the development of itch.

## 8. Conclusions

From the evidence discussed above, it is evident that the TRPV4 channel plays important roles in cell and organismal physiology. The last decade has witnessed an increased explosion of evidence on the role of this protein in several diseases. Consequently, synthetic agonists and antagonists of this protein have been designed, have been tested, or are currently being examined for their safety in the treatment of human pathologies.

The pivotal role that the TRPV4 channel plays in regulating lung and vascular physiology has established this channel as a possible therapeutic target for the treatment of some pulmonary affections. This has prompted the design of clinicals trials to evaluate the effects of the GSK2798745 molecule, a specific TRPV4 inhibitor, in some conditions such as heart failure, pulmonary edema and cough. To date, there are six clinical assays registered in the ClinicalTrials.gov website and five of them have been completed and have shown that this inhibitor is a molecule that may be of use in the pharmaceutical field. 

For example, the effects of GSK2798745 have been assayed in patients with heart failure (ClinicalTrial NCT02497937 in phase 2). A parameter used to evaluate the progression of this disease is to determine the degree of acute pulmonary congestion through the establishment of the Diffusing Capacity of the Lung for Carbon Monoxide (DLco). This clinical assay involved the study of patients with heart failure classified as placebo and GSK2798745-treated groups. After 14 days of the assay, the patients treated with GSK2798745 showed an increase in their DLco, indicating that TRPV4 inhibition promotes the recovery of pulmonary gas transfer.

Furthermore, the antitussive effect of the GSK2798745 has also been evaluated in patients with chronic cough (ClinicalTrials, Identifier: NCT03372603) where a positive effect of this compound was not observed in the relief of cough, suggesting that the block of TRPV4 function is not an alternative that results in antitussive effects.

Likewise, the effects of GSK2798745 have also been investigated in pulmonary vascular permeability, which is altered in ARDS [115]. This was studied in a clinical trial using a model of LPS induced lung inflammation in healthy volunteers (ClinicalTrials, Identifier: NCT03511105). Bronchoalveolar lavage (BAL) samples were collected to determine changes in barrier permeability and inflammation, before and after the LPS treatment, respectively. BAL samples from patients co-treated with LPS and GSK2798745 displayed small reductions in total protein levels and neutrophils counts, as compared to the placebo group.

Finally, it was recently reported that GSK2798745 is well-tolerated when this compound is systemically applied in healthy subjects and in patients with heart failure, indicating the safety and tolerability of this TRPV4 blocker (ClinicalTrials, Identifier: NCT02119260) [151].

The importance of this ion channel has even been laid out for strong consideration during the actual COVID-19 pandemic, as modulation of its function in the lungs may improve the outcome of patients [151]. This is of particular importance since it is of equal importance to find both: a vaccine and a treatment for this disease. We have acquired a large body of knowledge on how TRPV4 functions; nonetheless, it is still necessary to further investigate the roles of this channel in cell physiology. One example of this is that fewer endogenous agonists for TRPV4 have been described than, for example, the TRPV1 channel. Future studies will surely unveil interesting details of the molecular function of this amazing protein
